# A Mixed-Methods Analysis of Negative Patient Experiences in Emergency Department Care: Identifying Challenges and Evidence-Informed Strategies Across the Care Continuum

**DOI:** 10.1177/23743735251323795

**Published:** 2025-03-02

**Authors:** J. K. Fujioka, M. Walker, D. Rajab, S. A. Bartels

**Affiliations:** 1 Department of Medicine, Queen’s University, Kingston, ON, Canada; 2 Department of Emergency Medicine, Queen’s University, Kingston, ON, Canada; 3 Department of Public Health Sciences, Queen’s University, Kingston, ON, Canada

**Keywords:** emergency medicine, patient experience, patient perspectives/narratives, patient satisfaction, quality improvement

## Abstract

This mixed-methods study explores negative patient experiences within emergency departments (EDs), aiming to uncover systemic challenges and propose evidence-informed solutions. Of 2114 shared ED experiences, 306 (14.5%) were reported as "bad" or "very bad." Younger age, Indigenous status, financial instability, mental health disabilities, and non-heteronormative sexual identities were associated with negative ED experiences. Our research highlights key issues across the ED care continuum. During triage and registration, patients felt judged and perceived that their health concerns were under prioritized. Prolonged wait times contributed to feelings of neglect. During assessments, privacy concerns and lack of communication were prominent. Perceptions of misdiagnosis and stigmatization emerged as major concerns during the diagnosis and treatment phases. At discharge, insufficient follow-up and unclear instructions were frequently reported. Our findings underscore the need for improved communication, enhanced training to reduce stigma, and multi-pronged strategies to address the root causes of patient dissatisfaction. These insights can guide healthcare practitioners and policymakers in fostering a more inclusive and supportive ED environment, ultimately improving patient experiences and outcomes.

## Introduction

Navigating the emergency department (ED) is often challenging for patients, marked by disparities in access, treatment, and outcomes across different populations.^1–3^ EDs are currently facing an unprecedented surge in demand due to resource limitations, primary care provider shortages, and the aftermath of COVID-19.^4^ The overburdened ED environment can impact the quality of patient care.^3,5,6^ Overcrowding, for example, leads to longer wait times and delayed care, impacting patient satisfaction and outcomes.^3^ Additionally, an overwhelmed ED negatively influences patient-clinician interactions by contributing to automatic cognitive processes among ED staff such as categorization, stereotyping, and implicit biases, which disproportionately impacts those who experience social disadvantage.^5^

Understanding negative ED experiences is crucial due to their potential impacts on patient behavior and outcomes. For example, negative experiences, such as prolonged wait times or perceived lack of healthcare provider empathy, can lead to delayed care-seeking or care avoidance,^7^ thus eroding trust in the healthcare system and potentially worsening health outcomes.^7,8^ Patients may also be less likely to follow care recommendations, contributing to suboptimal health. During COVID-19, delayed care-seeking contributed to disease spread and exacerbated underlying health conditions.^9^ Delayed care also incurs an economic burden, intensifying healthcare costs for higher acuity needs, and straining the system leading to provider burnout.^10^ Addressing these negative experiences is paramount to mitigate unfavorable consequences on patient outcomes and overall public health.

Existing research addresses ED experiences among specific minority populations, such as LGBTQ+, racialized populations and people who use substances, highlighting experiences of stigma and discrimination.^6,11,12^ However, a significant gap exists in understanding *why* individuals have negative experiences at distinct steps of the ED care pathway. Additionally, current studies are often limited in scope and narrowly concentrate on a single target population despite the need for comprehensive intersectional data. To bridge this knowledge gap, our mixed-methods study combined a large sample of quantitative survey data and qualitative narrative data to comprehensively explore negative experiences in the ED. Our research aim was to identify which aspects of the ED care continuum are perceived most negatively and why. Understanding these experiences provides a roadmap for optimizing resources, allowing EDs to handle increasing demand while maintaining care quality.

Our research utilized the patient journey map of ED care by Harvey et al as a foundational framework.^13^ By integrating and expanding upon its insights, we aimed to develop a comprehensive understanding of negative patient experiences in EDs. The insights from Harvey et al informed the identification of specific points along the care continuum where patients encountered issues, guiding our analysis and helping to pinpoint areas needing quality improvement.

## Methods

### Study Design

This study is part of a larger mixed-methods, cross-sectional study involving 2114 ED experiences shared from 1953 participants. We used a sensemaking approach, which empowers participants to share and self-interpret their experiences, through qualitative and quantitative survey data, respectfully.^14^

### Study Setting and Participants

Participants were recruited from the ED and Urgent Care Centre (UCC) at the Kingston Health Sciences Centre (KHSC) and at community service agencies in Kingston, Ontario, Canada from June to August 2021. KHSC is a large tertiary hospital that serves a catchment of 500,000 people from surrounding urban and rural communities within southeastern Ontario, Canada.^15^ Trained Research Assistants (RAs) approached eligible participants in person at the ED and UCC during study hours (9:00 am to 9:00 pm, Monday to Friday) to invite them to participate in the study. Additionally, posters and flyers with contact information were placed in these recruitment areas to facilitate participant engagement and allow individuals to express interest independently. Eligible participants included medically stable patients aged 16 and older with sufficient English fluency. Recruitment took place at both the ED and UCC since both sites are staffed by the same physician group and are part of KHSC. Including both sites was deemed important to augment diversity among the sample and improve generalizability.

To include individuals not actively seeking care in the ED or UCC during the study period, RAs also recruited eligible participants from community organizations, such as Home-Based Housing, St. Vincent de Paul Society of Kingston, and the Integrated Care Hub.

### Data Collection

Participants used Spryng.io software on handheld tablets to share a typed or audio-recorded ED experience, termed “micronarrative,” that had occurred within the previous 24 months. These micronarratives were then self-analyzed through a series of multiple-choice questions that required participants to further contextualize their experiences, including rating their overall experience on a Likert scale from “very bad” to “very good.” Multiple-choice questions also gathered sociodemographic information. The survey, developed with community partners, took about 15 min to complete and participants could share multiple ED experiences.

### Data Analysis

Descriptive statistics, including frequencies and proportions (%), characterized the study sample and were disaggregated by positive and negative experiences as well as by various aspects of the ED care pathway. Chi-squared tests were used to determine whether there was a statistically significant difference (P < 0.05) between sociodemographic variables and reported ED care experiences. All quantitative data were analyzed using SPSS (IBM SPSS Statistics V.26.0.0.0).

Micronarratives of participants who reported their ED experience as a “bad” or “very bad” were qualitatively analyzed to better understand negative experiences. Qualitative data was transcribed verbatim and analyzed using inductive thematic analysis to identify emergent themes. Following Braun and Clarke's framework,^16^ two researchers (JKF, DR) independently analyzed an initial set of 50 micronarratives to develop a preliminary coding framework. This independent coding ensured that multiple perspectives informed the initial interpretations. The researchers then met to refine the codebook, discussing codes and emerging themes to resolve discrepancies through consensus. Using the finalized codebook, the framework was applied to the remaining micronarrative data. Subthemes and overarching themes were iteratively developed through collaborative discussions among the research team, ensuring that the final thematic structure was robust and reflective of the data.

Using a mixed-methods approach, the qualitative findings contextualized the quantitative data to increase depth and understanding. Integration of quantitative survey and qualitative micronarrative data occurred during interpretation, employing triangulation to achieve a more holistic understanding of the elements influencing adverse experiences along the ED care journey. To validate findings and ensure they aligned with community perspectives, the analyzed data was cross-referenced with retrospective focus group data exploring the ED experiences of specific populations.

### Use of Harvey et al.'s Patient Journey Map Framework

Our research utilized the patient journey map of ED care by Harvey et al as a foundational framework.^13^ This journey map was developed through a multi-method approach, incorporating insights from patient interviews, surveys, journey mapping workshops, staff interviews, and ethnographic observations across multiple EDs in Alberta. The map outlines key stages of the ED care process, including triage, registration, assessment, treatment, reassessment, and decision-making, highlighting both patient experiences and challenges at each stage. It emphasizes the impact of communication, physical layout, wayfinding, and staffing on patient experiences. The framework also identifies recurring issues, such as unclear processes, inadequate signage, and privacy concerns, that contribute to negative patient perceptions and stress.

By integrating and expanding upon its insights, we aimed to develop a comprehensive understanding of negative patient experiences in EDs. Specifically, we built on Harvey et al's framework to help organize our data into key themes across the ED care journey. Insights from Harvey et al were instrumental in pinpointing specific areas along the care continuum where patients experienced challenges. These insights guided our analysis and highlighted key areas requiring quality improvement.

### Ethical Considerations

This study received approval from the Queen's University Health Sciences and Affiliated Teaching Hospitals Research Ethics Board (#6029400). Informed consent was obtained from all participants before they completed the survey. No identifiable information was collected; data were anonymous from the time of collection. Participants received a $5 coffee gift card as a token of appreciation for participation.

## Results

Among 2114 ED micronarratives, 306 (14.5%) were reported to be a “bad” or “very bad” ED experience while 976 (46.2%) were reported to be a “good” or “very good” experience.

Demographic data are summarized in Table 1. Participants’ overall feelings about their ED visit were statistically different between age groups (P < 0.001) with older patients (>65 years old) reporting more positive experiences and younger patients (<18 years old) reporting more negative experiences. Patient experience was found to differ significantly across ethnic groups (P = 0.03), with variation in reported experiences among White/European, East Asian/Southeast Asian, and Indigenous-identifying patients. Financial struggles were also associated with differences in patient experiences (P < 0.001), with those who never struggled financially reporting a higher proportion of positive experiences compared to those who constantly struggled financially. Disability status showed a significant relationship with patient experience (P < 0.001), as individuals without disabilities reported more positive experiences overall, while those with mental health disabilities had a higher proportion of negative encounters. Lastly, sexual orientation was significantly associated with patient experiences (P < 0.001), with straight patients more frequently reporting positive experiences compared to bisexual and pansexual individuals.

**Table 1.** Participant Sociodemographic Characteristics Disaggregated by Patients’ Feelings About Their ED Visit.

Demographic variable		Bad/Very bad % (n); N = 306	Good/Very good % (n); N = 976	P-value
Patient age	<18 years old	6.9% (21)	9.8% (96)	<0.001
	18–25 years old	23.2% (71)	14.9% (145)	
	26–45 years old	35.0% (107)	28.3% (276)	
	46–65 years old	22.5% (69)	26.2% (256)	
	>65 years old	10.1% (31)	20.0% (195)	
	Not sure/prefer not to say	2.3% (7)	0.8% (8)	
Gender identity	Woman	56.7% (173)	52.6% (512)	0.091
	Man	38.7% (118)	45.0% (438)	
	Non-binary	2.3% (7)	1.3% (13)	
	Prefer not to say	2.3% (7)	1.1% (11)	
Ethnicity	White/European	74.8% (228)	79.8% (772)	0.028
	Indigenous	10.2% (31)	5.6% (54)	
	Black	1.6% (5)	1.8% (17)	
	South Asian	1.3% (4)	1.6% (15)	
	East Asian/Southeast Asian	0.3% (1)	2.6% (25)	
	Latin American	1.6% (5)	1.0% (10)	
	West Asian	0.7% (2)	0.7% (7)	
	Arab	0.7% (2)	0.3% (3)	
	One or more ethnicity	4.3% (13)	2.3% (22)	
	Not sure/prefer not to say	4.6% (14)	4.3% (42)	
Struggle to make ends meet	Never	22.2% (68)	48.1% (469)	<0.001
	Rarely	14.7% (45)	16.0% (156)	
	Sometimes	19.6% (60)	14.0% (137)	
	Often	12.4% (38)	6.7% (65)	
	All the time	24.5% (75)	8.0% (78)	
	Not sure/prefer not to say	6.5% (20)	7.3% (71)	
Disability	No disability	39.9% (122)	65.2% (636)	<0.001
	Mental health disability	26.8% (82)	9.9% (97)	
	Physical disability	14.1% (43)	10.6% (103)	
	Intellectual disability	2.3% (7)	1.3% (13)	
	Hearing loss/deafness	1.3% (4)	1.9% (19)	
	Low vision/blindness	0.7% (2)	0.2% (2)	
	Other (learning disability, autism spectrum disorder, neurodiverse…)	4.6% (14)	3.1% (30)	
	Not sure/prefer not to say	10.5% (32)	7.8% (76)	
Sexual identity	Straight	69.9% (214)	83.3% (813)	<0.001
	Bisexual	12.7% (39)	5.2% (51)	
	Pansexual	3.9% (12)	1.1% (11)	
	Gay/Lesbian	3.3% (10)	2.2% (21)	
	Questioning/unsure	1.6% (5)	0.3% (3)	
	Asexual	0.7% (2)	0.3% (3)	
	Sexual identity not on this list	0.7% (2)	0.5% (5)	
	Not sure/prefer not to say	7.2% (22)	7.1% (69)	

Abbreviation: ED, emergency department.

Table 2 presents the distribution of participants’ self-reported “bad" or "very bad" ED experiences across stages of the ED care pathway. Among these 306 micronarratives (14.5%), the most frequently cited areas of dissatisfaction were with doctors (32.7%), nursing staff (19.0%), and the waiting room (16.0%).

**Table 2.** Distribution of Negative Experiences in EDs by Specific Components of ED Care.

	“Bad” or “Very Bad” overall experience	
Events of the story were focused on…	N (/306)	%
Doctors	100	32.7%
Nursing staff	58	19.0%
Waiting room	49	16.0%
Triage	33	10.8%
Security officers	9	2.9%
Social workers	6	2.0%
Triage	33	10.8%
Registration	2	0.7%
Other^a^	13	4.2%
Not sure/prefer not to say	24	7.8%
Total	306	100%

^a^ The "Other" category included experiences related to trainees, interactions involving all staff, and issues with equipment such as stretchers.

Abbreviation: ED, emergency department.

Subsequent sections provide detailed insights into the negative patient experiences at each of these phases of care.

### Triage and Registration

The ED triage and registration process contributed to negative experiences, with 11.5% expressing dissatisfaction at this stage. Patients perceived biases, often due to feeling unfairly judged for potential drug-seeking behaviors based on their outward appearance and past mental health and substance use challenges. Some shared that triage and registration staff failed to believe in the legitimacy of their health concerns, leading to a lack of recognition of urgency. Sensitivity and privacy concerns arose when patients were questioned in the public intake area. One patient reported being turned away due to a lack of identification stemming from being unhoused.

### Waiting Area

Sixteen percent of micronarratives expressed negative experiences in the waiting area, specifically discontent with wait times. Several participants described prolonged wait times in chaotic situations leading to an overwhelming sense of being forgotten and resultant anxiety. Other participants faced aggressive interactions with security staff, with some describing physical assault during these encounters. Beyond wait times and security concerns, patients also voiced concerns related to exposure to infectious contacts in the crowded waiting area.

### Assessment and Investigations

Overall, 32.7% of patients expressed dissatisfaction with doctors, while 19.0% were discontent with nursing staff, and 2.3% had concerns about porters, X-ray, or CT technicians. Participants highlighted worries about privacy and dignity, a lack of explanations and informed consent, and limited physical examinations. Several patients were dissatisfied with both the outcomes of their assessments and the communication received from medical professionals. For instance, one participant reported having to actively seek more information and perceived that their care providers were dismissive of their concerns. Similar sentiments were echoed in cases where participants perceived that a physician's approach was apathetic, with participants raising concerns about potential misdiagnoses. Another participant expressed frustration with the perceived absence of comprehensive testing, feeling repeatedly overlooked. Others described a lack of explanation and informed consent for the various tests and investigations performed. These narratives emphasized the critical need for improved communication, thorough examinations, and a patient-centric approach during ED assessments and investigations.

### Diagnosis and Treatment

During diagnosis and treatment, patients expressed challenges such as inadequate support for mental health, difficulties managing chronic illnesses and pain, and the impact of stigma leading to misdiagnoses and diagnostic errors. One common concern was the perception of being unjustly labeled as "pill seekers" when seeking pain management, often due to a lack of understanding of their conditions. One participant faced dismissal and misdiagnosis due to behavioral challenges, leading to feelings of inadequate care. In another case, a delayed diagnosis resulted in a child's fractured arm being discovered days later. Instances of cancer misdiagnosis, inadequate attention to severe conditions, and mismanagement of pain further underscored patients’ perceived inadequacies. A particularly alarming case involved a patient with chest pain whose pericarditis was initially dismissed as an anxiety attack, emphasizing the need for thorough patient assessments.

### Discharge

Concerning discharge, 1.6% of individuals reported negative experiences due to insufficient follow-up and referrals, inadequate mental health support, neglect of social needs, reliance on short-term solutions, and a general lack of clear discharge instructions. Patient micronarratives emphasized an overarching sense that the ED care had not addressed their underlying needs. Some participants perceived they were discharged into vulnerable situations. For instance, one unhoused patient detailed being found in distress after discharge during a rainstorm. Another patient experiencing homelessness, lacked accommodations for recovery from their illness. Additionally, an individual shared that they suffered a second heart attack shortly after a perceived premature discharge, highlighting the critical repercussions of inadequate post-discharge care.

### Predominant Themes Across the continuum of ED Care

**Figure 1.**
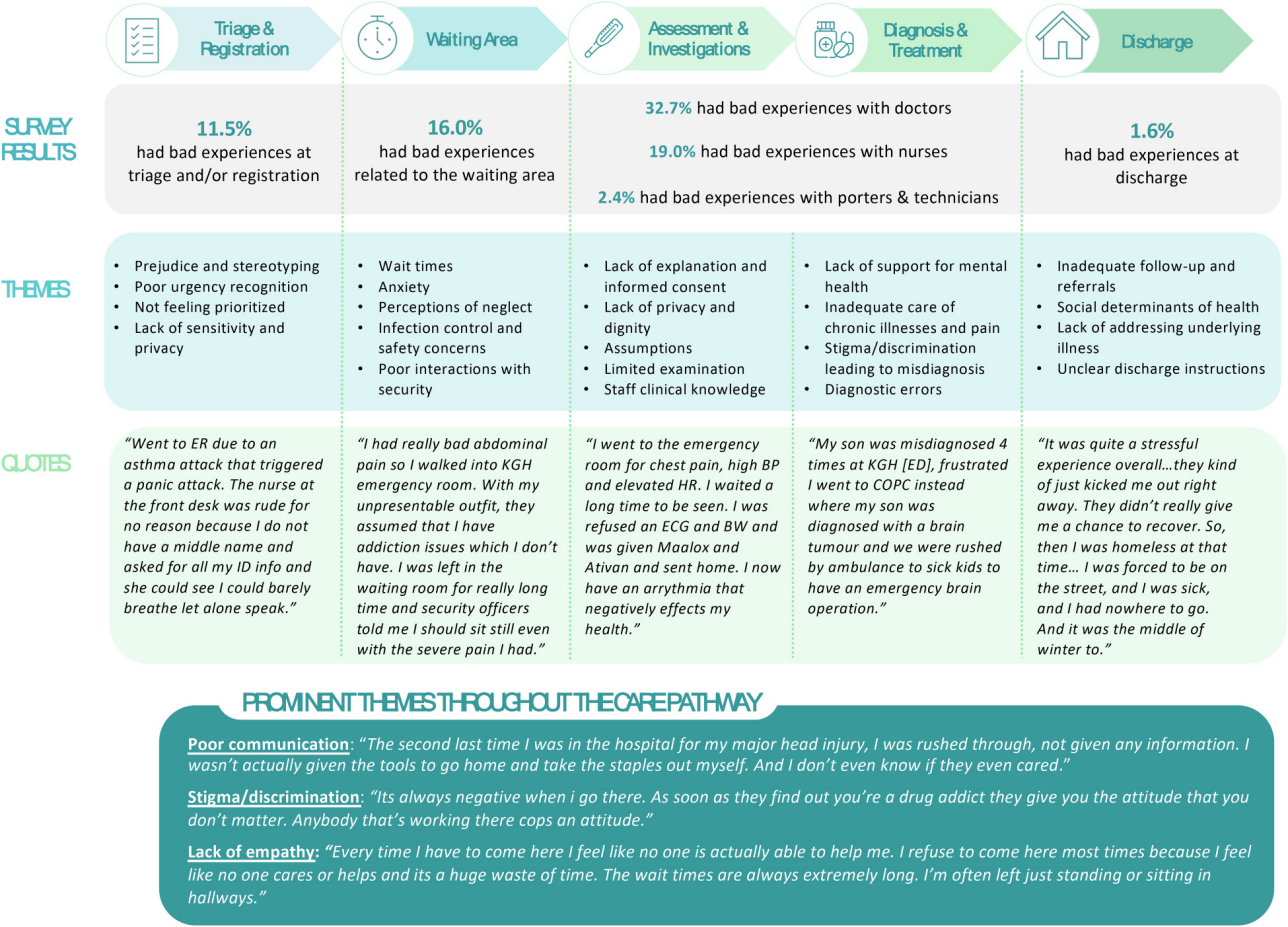
Negative experiences perceived by patients across the ED care continuum. Abbreviation: ED, emergency department.

Several themes emerged consistently across the ED care continuum. For instance, many patients reported poor communication from staff and receiving insufficient information throughout their ED journey. Similarly, language barriers exacerbated negative experiences across all aspects of ED care. Some individuals with special needs such as autism faced additional challenges and reported a lack of accommodations across their ED visits. Experiences of stigma and discrimination were described throughout the ED care continuum, often based on a history of substance use. Several participants noted that once healthcare providers learned of their drug use history, their treatment noticeably shifted, often becoming disrespectful and judgmental. Some felt dehumanized, unwelcomed, and, in some cases, accused of drug-seeking behavior, creating a pervasive sense of mistreatment. Furthermore, some participants noted a significant lack of empathy from staff, marked by dismissive attitudes, prolonged wait times without adequate assistance, and insufficient responses to severe mental health conditions. This contributed to feelings of helplessness and frustration with several patents expressing a reluctance to return the ED due to the perceived lack of compassionate care (Figure 1).

## Discussion

Our study examined negative ED experiences among a large sample of diverse patients, revealing challenges that extend across the care journey. Findings suggest that younger age, Indigenous status, financial instability, mental health disabilities, and non-heteronormative sexual identities were associated with negative ED experiences. Qualitative data shed light on areas for improvement.

During triage and registration, perceived biases and unfair judgments, especially related to drug-seeking behavior, significantly impacted patient satisfaction. This echoes findings from prior research that highlighted cognitive biases among emergency triage nurses. For instance, one study identified numerous cognitive biases influencing triage nurse decision-making when prioritizing patients.^17^ Staff training and sensitization to reduce biases and improve awareness may enhance patient experiences during triage. Alternatively, implementing electronic kiosks for ED triage may ameliorate the risk of bias and address privacy concerns. Research on the effectiveness of kiosks in EDs has consistently shown positive outcomes, such as decreased wait times, enhanced identification processes, and expedited data entry.^18^

Identified waiting area experiences also resonate with existing literature on patient satisfaction.^19,20^ For example, Nyce et al found a significant association between extended door-to-physician time and poorer patient experience, particularly among discharged patients and those with hospital stays ≤4 days.^20^ Furthermore, prolonged ED wait times have been linked to less favorable patient experiences. Research suggests several strategies to improve wait times in the ED such as implementing advanced triage systems,^21^ enhancing communication,^22^ employing telemedicine for remote assessments,^23,24^ streamlining diagnostic procedures, and utilizing predictive analytics to forecast patient influx and resource allocation more efficiently.^25,26^ Additionally, introducing lean management principles and increasing staffing levels during peak hours have shown promise in reducing wait times and enhancing overall ED patient experiences.^27,28^

During the assessment and investigations phase of ED care, our study highlighted the significance of privacy, informed consent, and clear communication in improving patient satisfaction. Participants noted issues with misdiagnosis and perceived unmet needs. A study by Geraloma et al identified three key patient needs with respect to testing and diagnosis: an explanation for symptoms, treatment guidance, and clear communication about investigations, treatment, and diagnosis.^22^ While some patients sought further guidance in the absence of a diagnosis, others were content not to have a diagnosis. Effective symptom management and communication with providers were prioritized. Suggested improvements for diagnostic accuracy include the use of decision aids, fostering teamwork, involving patients in decision-making, structured learning from mistakes, and establishing protocols for test follow-up.^29^ Enhancing clinicians’ proficiency to communicate diagnostic uncertainty can also improve physician-patient interactions and help to manage patient expectations.^30^

Concerns raised related to discharge align with earlier studies that emphasized the need for clear discharge instructions, comprehensive follow-up plans, and tailored support for vulnerable populations.^22^ A meta-analysis found that while verbal discharge instructions have low recall rates (as low as 8%), adding written information improved recall to 47% to 58%.^31^ Given time constraints in busy EDs, alternative strategies like online resources and handouts can efficiently supplement verbal communication. Our study also emphasizes the imperative to enhance continuity of care and community supports for individuals made vulnerable due to substance use, mental health issues, and/or housing instability. Integrated care pathways, such as patient navigators, partnerships with community organizations, and integration of telehealth, can be crucial for aligning acute care requirements with comprehensive community supports.^32-34^ Achieving this requires community education, behavioral health services, and strong collaboration between the ED and community organizations.

Participants frequently reported poor communication, lack of information, language barriers, and stigma, particularly related to substance use history, during their ED encounters. Addressing these issues requires enhanced staff training, greater sensitivity to diverse patient needs, and fostering a more empathetic, non-judgmental environment. Our study emphasizes that patients prefer clear communication about triage, wait times, tests, diagnoses, and treatment in understandable, non-medical language. Studies suggest healthcare providers should avoid patronizing language and improve coordination to minimize redundant questioning, especially when patients are unwell.^8^ Addressing provider burnout and overcrowding may bolster staff capacity to compassionately respond to patient concerns. However, education, tranquil staff spaces, and counseling have not significantly reduced compassion fatigue, highlighting the challenges of the demanding ED environment.^35^

## Future Directions

While perceived biases during triage and prolonged wait times significantly influence patient satisfaction and outcomes, it's essential to recognize wider systemic issues contributing to negative ED care experiences. We recognize that the root causes of patient frustration often lie in the failing healthcare system more broadly rather than solely on the care provided by specific ED providers and/or on the burden of COVID-19. For instance, patients’ reliance on the ED for non-emergent conditions underscores the urgent need for more accessible primary care and specialist services. The lack of affordable housing and shelter systems further exacerbates the situation by leaving some patients with no choice but to seek help in the ED.^36^ While supporting staff in providing patient-centered care is crucial, addressing underlying systemic issues is equally imperative. Integrating comprehensive community supports with acute care pathways is essential for ensuring seamless transitions and sustained assistance beyond the ED. However, it's evident that addressing these challenges requires systemic changes, including addressing primary care shortages and ED overcrowding.

## Strengths and Limitations

This study is not without limitations, including the absence of perspectives from ED healthcare providers, indicating the need for future research to incorporate their views for comparative analysis. The reliance on a convenience sample during specific study hours may limit the representation of the Kingston community, affecting the generalizability of findings to other patient populations and locations. Additionally, because the survey intentionally excluded any identifying information about patients or providers, we cannot assess the quality of care delivered. While the perspectives shared are valuable and reflect patients’ lived experiences, which are important in their own right, they may not offer a complete picture of the care provided. This limitation underscores the need to interpret these perceptions as one dimension of understanding healthcare delivery. Finally, we recruited participants from both an UCC and an ED, and it is noteworthy that the patients’ choice of where to seek care may have influenced their expectations.

Nevertheless, the study exhibits several strengths, such as collective perspectives from a diverse population of participants. The sensemaking approach empowered participants to highlight aspects of ED care most significant to them, and data collection at community organizations reduced selection bias by engaging those with prior negative ED experiences who may no longer be seeking ED care. Further, the sensemaking approach minimized interviewer and social desirability biases, and the large sample size enhances the study's reliability and generalizability.

## Conclusion

This study revealed a variety of challenges along the ED care continuum, underscoring key areas for quality improvement. Poor communication, prolonged wait times, and stigma and discrimination are recurrent issues that detrimentally impact patient experiences. Addressing these challenges requires enhancing empathy, implementing advanced triage systems, and fostering a more inclusive and supportive environment for all patients, especially those from marginalized communities. Furthermore, systemic issues such as healthcare resource limitations and primary care shortages must be tackled to reduce ED overcrowding and improve patient care pathways. By prioritizing patient-centered strategies and comprehensive community support integration, EDs can enhance the overall patient experience, leading to better health outcomes and increased trust in healthcare systems​.
